# Antismoking Campaigns' Perception and Gender Differences: A Comparison among EEG Indices

**DOI:** 10.1155/2019/7348795

**Published:** 2019-04-17

**Authors:** Giulia Cartocci, Enrica Modica, Dario Rossi, Bianca M. S. Inguscio, Pietro Aricò, Ana C. Martinez Levy, Marco Mancini, Patrizia Cherubino, Fabio Babiloni

**Affiliations:** ^1^Department of Molecular Medicine, Sapienza University of Rome, Rome, Italy; ^2^BrainSigns Srl, Via Sesto Celere, 00152 Rome, Italy; ^3^Dept. Anatomical, Histological, Forensic & Orthopedic Sciences, Sapienza University of Rome, Rome, Italy; ^4^IRCCS Fondazione Santa Lucia, Neuroelectrical Imaging and BCI Lab, Via Ardeatina, 306, 00179 Rome, Italy; ^5^Department of Communication, Social Research, Sapienza University of Rome, Via Salaria 113, 00198 Rome, Italy; ^6^Department of Computer Science, Hangzhou Dianzi University, Hangzhou, China

## Abstract

Human factors' aim is to understand and evaluate the interactions between people and tasks, technologies, and environment. Among human factors, it is possible then to include the subjective reaction to external stimuli, due to individual's characteristics and states of mind. These processes are also involved in the perception of antismoking public service announcements (PSAs), the main tool for governments to contrast the first cause of preventable deaths in the world: tobacco addiction. In the light of that, in the present article, it has been investigated through the comparison of different electroencephalographic (EEG) indices a typical item known to be able of influencing PSA perception, that is gender. In order to investigate the neurophysiological underpinnings of such different perception, we tested two PSAs: one with a female character and one with a male character. Furthermore, the experimental sample was divided into men and women, as well as smokers and nonsmokers. The employed EEG indices were the mental engagement (ME: the ratio between beta activity and the sum of alpha and theta activity); the approach/withdrawal (AW: the frontal alpha asymmetry in the alpha band); and the frontal theta activity and the spectral asymmetry index (SASI: the ratio between beta minus theta and beta plus theta). Results suggested that the ME and the AW presented an opposite trend, with smokers showing higher ME and lower AW than nonsmokers. The ME and the frontal theta also evidenced a statistically significant interaction between the kind of the PSA and the gender of the observers; specifically, women showed higher ME and frontal theta activity for the male character PSA. This study then supports the usefulness of the ME and frontal theta for purposes of PSAs targeting on the basis of gender issues and of the ME and the AW and for purposes of PSAs targeting on the basis of smoking habits.

## 1. Introduction

Smoking is the first cause of preventable deaths in the world [1]. It has been proved that tobacco companies specifically target women and children through their advertising, marketing, and promotion activities [2]. The main tool that governments can use to contrast such global emergency is constituted by antismoking public service announcements (PSAs). In relation to this, the possible gender differences in the response to antismoking measures, such as antismoking PSAs, are extremely worthy to be investigated. It has been previously highlighted how men and women respond differently to advertising [3, 4] and in particular to antismoking advertising [5–7]. In addition, it was seen a different modulation operated by the transportability within each sex [8] in response to advertising. Focusing on the gender effect in the reaction to smoking cue in antismoking advertisements, fMRI studies identified higher activation in women in several brain regions in comparison to male smokers, male exsmokers, and nonsmokers [6]. Furthermore, other fMRI research studies found a greater bilateral hippocampal/amygdala activation in males exposed to smoking cue in comparison to nonsmoking cues [7].

The mental engagement (ME) index was developed by Pope and colleagues [9] for its application in cognitive tasks, such as a closed-loop system to modulate task allocation. It was based on several evidences that the increases in beta activity would reflect a higher degree of alertness and greater engagement in the task, while increases in alpha and/or theta activity were supposed to reflect less alertness and decreased task engagement/information processing [10–16]. Concerning the ME application, for instance, it has been measured an improvement in performances during a vigilance task when this index was used as a criterion for switching between manual and automated piloting mode [17]. Subsequently, it has been applied to the assessment of the emotional influence on learning in an educational setting, resulting as predictive of the performance [18]. Also due to the emotional content often present in antismoking PSAs, we decided to apply such index to this study.

The approach/withdrawal (AW) index and the frontal theta index have been already applied to the study of antismoking PSAs, in relation to the investigation of neurophysiological correlates of effectiveness in such kind of advertising [19–22]. The AW was based on the theory of the frontal alpha asymmetry introduced by Davidson [23] and further adopted in several studies [24–27]. According to this theory, the prefrontal cortex (PFC) plays a crucial role in the circuitry that mediates both positive and negative motivation. In particular, various studies evidenced a relative increase in the left PFC activation in correspondence of the positive motivation, while an augmented right-sided anterior activation was observed during the negative motivation [28–32]. The application to advertising material of the frontal alpha asymmetry index, defined as the difference between the prefrontal right and left EEG power spectra alpha activity, has been already repeatedly reported [4, 33–36]. The frontal theta index was included among the assessed indices groups since its relation to the processing of the antismoking message of the PSA. In fact, higher values of frontal theta have been connected to higher levels of task difficulty [10, 37]. Moreover, it has been evidenced how the frontal theta activity represents a marker of cognitive processing during a visual task execution [11, 38]. The frontal theta as index of effort and processing has been already applied to a number of studies in different fields of research, such as neuroaesthetics [12, 39, 40]; advertising [40]; avionic [41–46] and car driving [16, 42, 47–49]; different challenging listening conditions both in normal hearing and in hearing impaired participants [50–52]; and human-computer interaction [53, 54] studies.

The spectral asymmetry index (SASI) was introduced by Hinrikus and colleagues with the aim of comparing the sensitivity of different electroencephalographic (EEG) indicators for the detection of depression [54]. Afterwards, it was applied to the reaction to emotional stimuli, providing evidence of the capability of discrimination of negative (SASI increase), positive (SASI decrease), and neutral stimuli [55]. SASI is based on the balance of EEG theta and beta frequency band powers, since evidences found a relation between EEG theta power and emotional activation. For example, Hu and colleagues [56] found evidence of a correlation between global theta power changes and “playfulness” emotions (amusement, interest, and joy) characterizing video stimuli. This evidence was also in accord with a previous study on videos by Aftanas and collaborators [57] who highlighted the major increase in theta power in response to video clips related to joy. On the other hand, beta power has been associated with high anxiety levels, especially in the right hemisphere [58], while decrease in beta activity with relaxation [59]. Therefore, SASI was expected to increase in the case of negatively perceived stimuli and to decrease in the case of positively perceived stimuli while compared to neutral pictures.

It is not clear how the gender and the previous smoking habit issues could interact with the observation of antismoking PSAs. Aim of this study was to compare the sensitivity of different EEG-based indices in detecting first gender issues and secondly smoking habit issues, in the reaction to antismoking PSAs. The final aim is to understand if such eventual cerebral patterns could be different in relation to some features of the employed PSAs in terms of characters and plot and in relation to some features of the experimental sample, that is, gender and tobacco use. If so, it could be explored in the future the possibility to evaluate the PSAs before their broadcast to understand if they could be successful (e.g., effective) or unsuccessful (e.g., ineffective) in generating appropriate healthy responses taking into account the particular gender and the smoking habit issues.

## 2. Materials and Methods

### 2.1. Participants

The experimental sample was composed of 46 participants (23 F and 23 M), divided in smokers (participants usually smoking at least 5 cigarettes per day) and nonsmokers. Smokers were 12 F and 11 M, while nonsmokers were 11 F and 12 M (Table 1). Participants were healthy volunteers aged between 25 and 55 years (mean age and standard deviation: F nonsmokers: 36.00 ± 12.05; F smokers: 36.25 ± 9.92; M nonsmokers: 33.50 ± 8.67; M smokers: 38.63 ± 10.58) and received a small compensation for their participation. All participants were given of detailed information about the study and signed informed consent. The experiment was performed in accord to the principles outlined in the Declaration of Helsinki of 1975, as revised in 2000, and it was approved by the Sapienza University of Rome Ethical Committee in charge for the Department of Molecular Medicine.

### 2.2. Protocol

**Figure 1:**
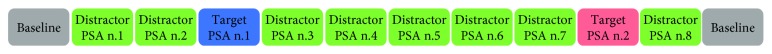
Structure of the protocol employed in the study. Each of the target stimuli could randomly appear in one of the ten possible PSA positions. Distractor stimuli were randomly placed in the remaining positions. At the beginning and at the end of the PSA train, there was the 1-minute baseline video.

During the execution of the test, participants were sitting on a comfortable chair in front of a computer screen, and they were not instructed with any particular task, just to be relaxed and to restrict head and body movements as much as possible. Participants were asked to watch a video composed by a train of ten randomly delivered antismoking PSAs, with a total length of 9 minutes, preceded and followed by a documentary (neutral in respect to gender; in fact it was about constellations) lasting 1 minute, and already used as baseline [19]. The target stimuli were two of the ten PSAs, and participants were exposed once to each stimulus (both target and distracting stimuli) (Figure 1):

- One male character antismoking PSA: CDC Roosevelt (USA, 2012–2015). The video displays a young man telling how he got a heart attack when he was just 45 years old and all the consequences of that event, beginning from the scar on his chest to the limitations in his everyday life, for instance, in climbing the stairs or playing with his son (retrievable at https://www.youtube.com/watch?v=OdmI35elnCQ).
- One female character antismoking PSA: Baby Love (Finland, 2013). The video displays a young pregnant woman at first apparently preparing the room for the baby she is waiting, but as long as the video develops, it turns out that she actually does horrible actions, like hanging knives instead of toys in the carillon over the cradle, or putting a snake into it. The video ends with the young woman lighting a cigarette and smoking with an ashtray placed on her pregnant belly (retrievable at https://www.youtube.com/watch?v=SPBQII5c9fw).

### 2.3. EEG Recordings and Signal Processing

For the recording of the EEG activity, it has been employed a portable 24-channel system (BEmicro, EBneuro, Italy) and an EEG frontal band with ten electrodes (Fpz, Fp1, Fp2, AFz, AF3, AF4, AF5, AF6, AF7, and AF8). Impedances were kept below 10 kΩ, and signals were acquired at a sampling rate of 256 Hz. EEG traces were digitally bandpass filtered by a 5^th^ order Butterworth filter ([2 ÷ 30] Hz), so as to reject the continuous component and high-frequency interferences.

At this point, the independent component analysis (ICA) was employed on cleaned EEG data, so as to identify and remove blinks activity contribution. For this purpose, the signal has been decomposed in 10 ICA components (the same number of EEG channels), but only the component related to blinks activity has been selected [60].

Moreover, specific procedures of the EEGLAB toolbox have been adopted [61], in order to remove further sources of artifacts (e.g., muscular activity and bioamplifier saturation). First of all, EEG signal has been segmented in epochs of 1 second, shifted of 0.5 seconds. Three epoch rejection criteria have been applied as follows:

- Threshold criterion: when amplitudes in the EEG signal were equal to or greater than 100 *μ*V, the corresponding epoch was labeled as artifact
- Trend criterion: in the aim to check the slope of the trend within the considered epochs, EEG epochs were interpolated, and if the slope of an epoch was higher than 10 *μ*V/s, it was labeled as artifact
- Sample-to-sample difference criterion: cases in which the amplitude difference between consecutive EEG samples was higher than 25 *μ*V, representing an abrupt nonphysiological variation, the corresponding EEG epoch was labeled as artifact

Specifically, all the adopted numeric values just mentioned were chosen accordingly to the guidelines reported in Delorme and Makeig [61].

After the application of the just mentioned epoch rejection criteria, in order to have an artifact-free EEG signal from which estimating the brain variations along the different conditions, all the EEG epochs labeled as artifact were rejected from the EEG dataset.

For each participant, the individual alpha frequency (IAF) was calculated over a 60-second long open eyes segment, recorded before the beginning of the experimental task. IAF was computed in order to define the EEG bands of interest according to the method suggested in the current scientific literature, i.e., each band is defined as “IAF ± *x*,” where IAF is the individual alpha frequency, in Hertz, and *x* an integer in the frequency domain [37]. Consequently, the EEG activity was divided in three main frequency bands: theta [IAF − 6 ÷ IAF − 2 Hz], alpha [IAF − 2 ÷ IAF + 2 Hz], and beta [IAF + 2 ÷ IAF + 14 Hz]. To summarize the activity of the cortical areas of interest in a specific frequency band, the global field power (GFP) [62] was then computed. The GFP summarizes the synchronization level of the brain activity over the scalp surface, and its measure corresponds to the spatial standard deviation. GFP estimates the quantity of activity at each time point in the field, simultaneously considering data from all recording electrodes, resulting in a reference-independent descriptor of the potential field [63]. The main concept of GFP is that scalp fields presenting high activity reflect the synchronous activation of a large number of intracranial neuronal elements, while fields with few field lines would contain little information. The GFP was already employed in studies of perceptual, attentional, and cognitive processing [64–66] as well as in clinical studies [67–69]. The GFP represents an index for the temporal determination of information from cognitive studies; moreover, it constitutes a parameter for the time-domain analysis of EEG (as in the present work), as it allows the identification of the maps of maximal electric field strength (hilliness).

Specifically, in this study, the GFP was calculated from a specific set of electrodes (the set depends on the investigated brain area, and for each index it will be specified later in the study) by performing the sum of squared values of EEG potential at each electrode, averaged for the number of involved electrodes, resulting in a time-varying waveform related to the increase or decrease of the global power in the analysed EEG. The GFP formula is specified in the following equation:

(1) $$\begin{array}{c} {\text{GFP}}_{\vartheta ,\text{Frontal}}=\frac{1}{N}\overset{N}{\underset{i=1}{\sum }}x_{\vartheta_{i}}{\left(t\right)}^{2}, \end{array}$$

where *ϑ* is the considered EEG band, *Frontal* is the considered cortical area, *N* is the number of electrodes included in the area of interest (in this example the Frontal area), *i* is the electrodes' index, and *x* represents the specific EEG sample at time (*t*), filtered within the related EEG band (i.e., *ϑ*) and for the specific channel *i*.

It was calculated then the average GFP value on all the GFP values estimated over 1 second of EEG signal. According to the following paragraphs, EEG indices were calculated and normalized for each second by using the mean and the standard deviation of the same neurometrics calculated on the baseline:

(2) $$\begin{array}{c} \text{normalized index}=\frac{\text{index}-{\text{mean}}_{\text{baseline}}}{\text{standard}{\text{ deviation}}_{\text{baseline}}}. \end{array}$$

### 2.4. Mental Engagement

The mental engagement (ME) index has been defined as the ratio between the activity in the beta band and the sum of alpha and theta activity (equation (3)), as defined by Pope and colleagues [9]:

(3) $$\begin{array}{c} \text{mental engagement index}=\frac{\text{GFP}\beta }{\text{GFP}\alpha +\text{GFP}\theta }. \end{array}$$

### 2.5. Approach Withdrawal

The approach/withdrawal (AW) index has been defined according to Davidson and colleagues [23] as the frontal alpha asymmetry as reported in the following formula:

(4) $$\begin{array}{c} \text{approach withdrawal index}=\text{GFP}\alpha \_\text{right}-\text{GFP}\alpha \_\text{left,} \end{array}$$

where the GFP*α*_right and GFP*α*_left stand for the GFP calculated among right (Fp2, AF4, and AF8) and left (Fp1, AF3, and AF7) electrodes, respectively, in the alpha (*α*) band. Higher AW values, reported by the subjects, stood for an approach motivation toward the stimulus, while lower AW values for a withdrawal motivation [70, 71].

### 2.6. Frontal Theta

The EEG activity in the theta band over all the frontal electrodes has been considered for the estimation of the mental effort/processing processes. An increase in the frontal theta (i.e., mental effort/processing) would imply an increase in the task difficulty [38, 49] and therefore in the attendance to the antismoking content of the PSA, as also already evidenced by studies in which the frontal theta activity has been investigated during the exposure to antismoking advertising [19–22].

### 2.7. Spectral Asymmetry Index

The spectral asymmetry index (SASI) has been defined by previous studies where it was calculated for each EEG electrode [54] and for groups of electrodes corresponding to cerebral regions [55], and here calculated, over all the frontal electrodes, according to the following equation:

(5) $$\begin{array}{c} \text{SASI}=\frac{\text{GFP}\beta -\text{GFP}\theta }{\text{GFP}\beta +\text{GFP}\theta }. \end{array}$$

### 2.8. Statistical Analysis

ANOVA test has been performed on all the considered indices. The first between-variable was the “Smoking Habit,” with 2 levels: smokers and nonsmokers; the second between-variable was the participants “Gender,” with two levels: men and women; the within variable was the “PSA kind,” with two levels: male character PSA and female character PSA. Duncan's post hoc comparisons have been performed on the statistically significant interactions. Logistic regression analysis was also performed on the indices, considering as dichotomic variables the participants' gender. Simple regression analysis has been performed in order to investigate the correlation between the number of smoked cigarettes by participants and the neurophysiological response indexed by the selected indices.

## 3. Results and Discussion

### 3.1. Mental Engagement Results

**Figure 2:**
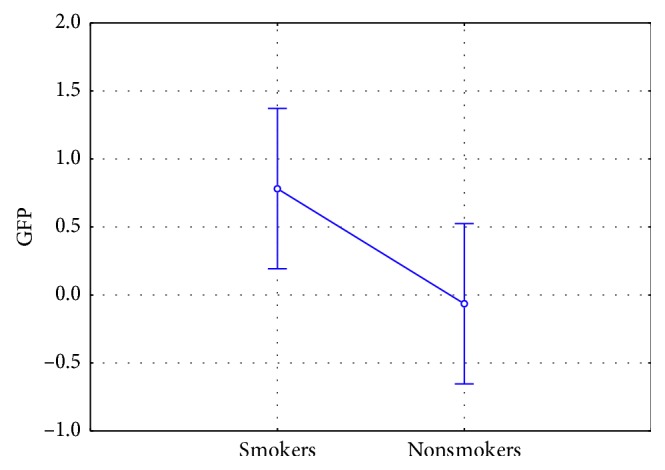
Mental engagement index: comparison among smokers and nonsmokers (*n*=46). Smokers showed a statistically significant increase in ME values in comparison to nonsmokers (F(1, 42) = 4.207, *p*=0.046). Vertical bars denote 0.95 confidence intervals.

**Figure 3:**
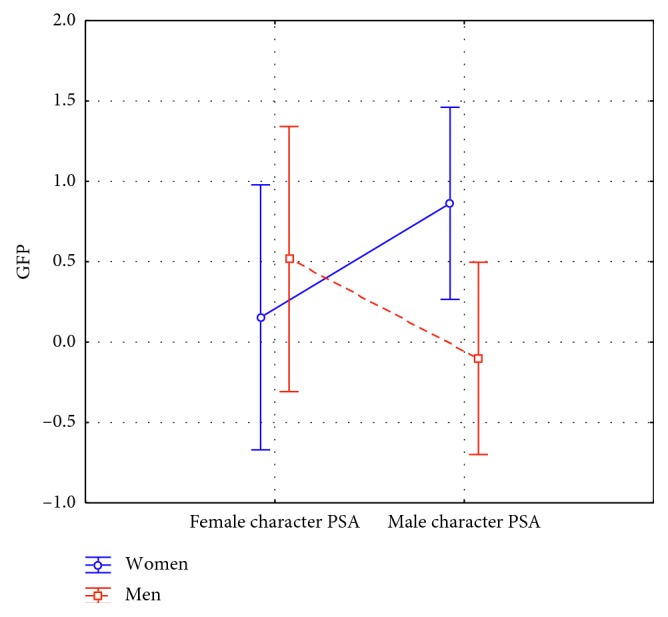
Mental engagement index: interaction between PSA kind and Gender (*n*=46). The statistical analysis reported a statistically significant interaction between the factor PSA and the factor Gender (F(1, 42) = 5.225, *p*=0.027). Post hoc analysis did not show any statistically significant difference in the pairwise comparisons. Vertical bars denote 0.95 confidence intervals.

Concerning the mental engagement (ME), results showed a statistically significant increase for ME values in smokers in comparison to nonsmokers (F(1, 42) = 4.207, *p*=0.046) (Figure 2). Furthermore, ANOVA test reported a statistically significant interaction between the factor PSA and the factor Gender (F(1, 42) = 5.225, *p*=0.027). Post hoc analysis did not show any statistically significant difference in the pairwise comparisons (Figure 3).

**Figure 4:**
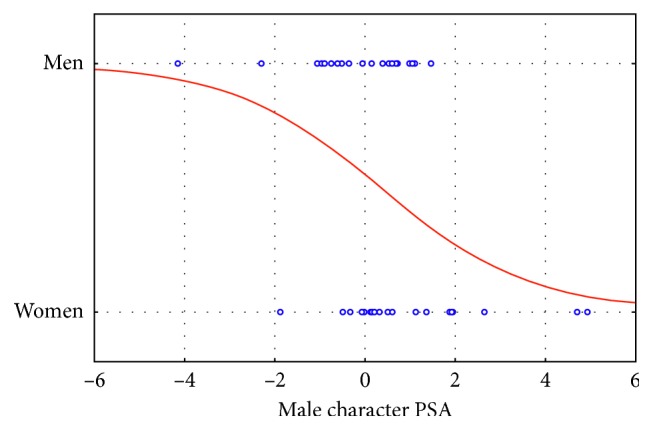
Mental engagement index: plot of the logistic regression between the male character PSA and Gender (*n*=46). The statistical analysis reported a statistical significance for such correlation (*p*=0.015) suggesting that men reported lower ME values in comparison to women in correspondence of the exposure to the male character PSA.

In addition, ME results evidenced a statistical significance in the logistic regression between the values obtained in response to the male character PSA and the Gender of participants (*p*=0.015) (Figure 4).

**Figure 5:**
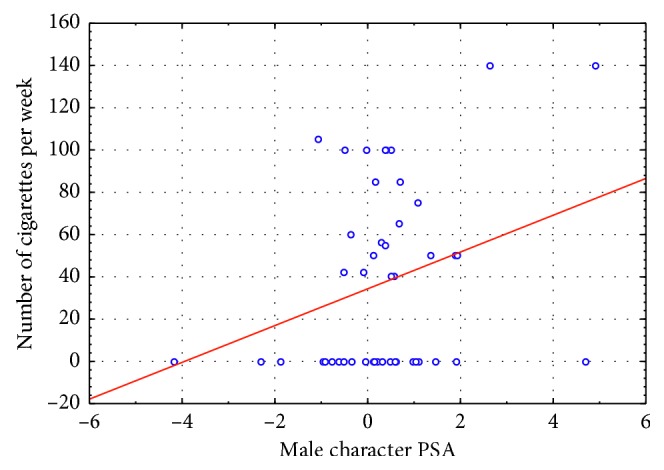
Mental engagement index: plot of the linear regression between the male character PSA and the number of cigarettes per week smoked by participants (*n*=46). The statistical analysis reported a statistical significance for such correlation (*p*=0.044) suggesting that heavy smokers reported higher ME values in correspondence of the exposure to the male character PSA.

Finally, ME results provided evidence of a correlation between the number of cigarettes per week smoked by participants and the ME values obtained in correspondence of the exposure to the male character PSA (*p*=0.044) (Figure 5).

### 3.2. Approach/Withdrawal Results

**Figure 6:**
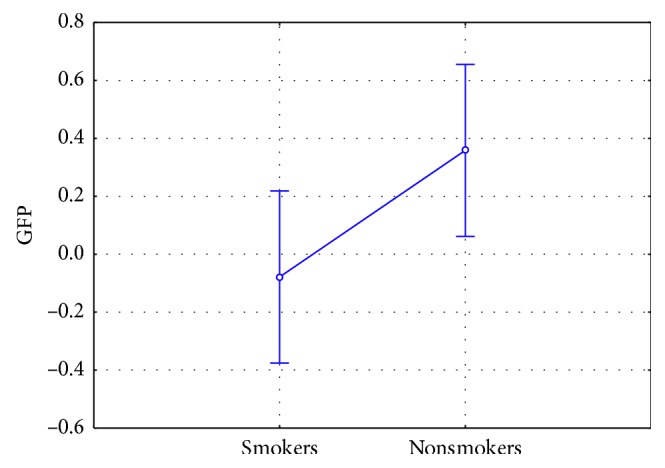
Approach/withdrawal index: comparison among smokers and nonsmokers (*n*=46). Nonsmokers showed a statistically significant increase in AW values in comparison to smokers (F(1, 42) = 4.413, *p*=0.042). Vertical bars denote 0.95 confidence intervals.

Results evidenced increased AW values for the nonsmokers in comparison to smokers (F(1, 42) = 4.413, *p*=0.042) (Figure 6). It is also interesting to note that nonsmokers presented positive AW values and therefore an approach tendency toward the antismoking PSAs, while smokers showed negative AW values and therefore a withdrawal tendency.

### 3.3. Frontal Theta Results

**Figure 7:**
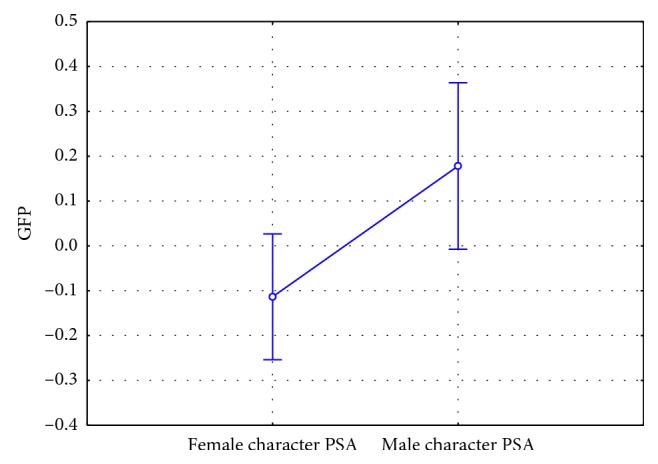
Frontal theta index: comparison between the kind of PSA (*n*=46). The male character PSA showed higher frontal theta values (F(1, 42) = 19.981, *p* < 0.001). Vertical bars denote 0.95 confidence intervals.

**Figure 8:**
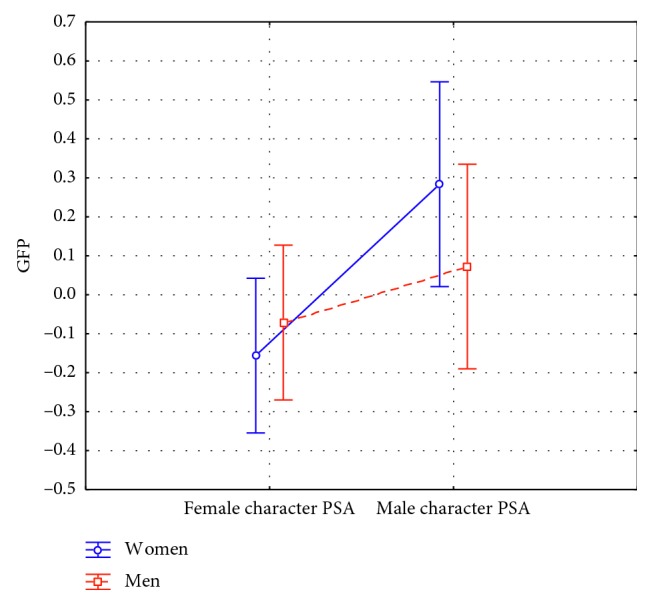
Frontal theta index: interaction between the kind of PSA and the Gender of the participants (F(1, 42) = 5.150, *p*=0.028) (*n*=46). The male character PSA showed higher frontal theta values in women in comparison to the female character PSA both in men (*p*=0.040) and women (*p* < 0.001). Vertical bars denote 0.95 confidence intervals.

Concerning frontal theta activity, it has been found a statistically significant effect for the kind of the PSA (F(1, 42) = 19.981, *p* < 0.001). In particular, the male character PSA showed higher frontal theta values (Figure 7). Furthermore, it has been found a statistically significant interaction between the variables PSA kind and Gender of the participants (F(1, 42) = 5.150, *p*=0.028) (Figure 8). The post hoc analysis highlighted also an increase of the frontal theta values reported by women participants in response to the male character PSA in comparison to the response of both women (*p* < 0.001) and men (*p*=0.040) to the exposure to the female character PSA.

### 3.4. Spectral Asymmetry Index Results

Concerning the SASI, the statistical analysis performed on the collected data did not provide any statistical significance.

## 4. Discussion

The higher ME values, the logistic regression results, and the higher frontal theta activity showed by women in response to the male character PSA (narrating the story of a man who had an heart attack at a very young age due to smoking) could be explained by the evidence that women in comparison to men have been found to be more influenced by advertisements that emphasize the negative effects of smoking on health [5].

The significant correlation between the number of cigarettes per week smoked by participants and the ME values stated that as long as the number of cigarettes increased, the relative ME values also increased. It is interesting to note that this was true only for the male character PSA, again, the one showing the negative health effects of smoking (heart attack and its consequences on the everyday life). This would be explained by the fact that smokers would feel more involved by such content, possibly resulting in higher ME values.

The tendency of approach showed by nonsmokers during the exposure to the antismoking PSAs could be explained by the perceived higher effectiveness by nonsmokers in response to advertisements eliciting strong negative emotions (sadness and fear) [72], like the PSAs included in this study.

The higher frontal theta levels reported for the male character PSA in comparison to the female character PSA were probably due to the complexity of the narrated story, in accord to a previous study providing evidence that the presence of a narrative structure in video commercials resulted in higher theta power of the left frontal area [73].

The lack of significant results obtained by the analysis employing the SASI could be explained by the fact that both PSAs would be negatively perceived due to the frightening and worrying nature of their content. However, it must also be considered the method of investigating the SASI index at the level of a region in the present article and not at the level of each single electrode or small area (grouping the electrodes by 3), as in previous studies [54, 55].

## 5. Conclusions

This study, through the comparison among EEG indices, showed the sensitivity of the ME and the frontal theta index in evidencing gender influences (both of the PSA characters and of the participants) on the neurophysiological response. The ME presented also the advantage of pointing out also a difference based on the smoking habit of the participants, an aspect highlighted also by the AW index. The frontal theta on the other hand highlighted the significant effect of the PSA character gender. This study then supports the usefulness of the ME and frontal theta for purposes of PSAs targeting on the basis of gender issues and of the ME and the AW and for purposes of PSAs targeting on the basis of smoking habits.

## Figures and Tables

**Table 1:** The table reports the number of cigarettes per week smoked by participants.

Female participants	Cigarettes/week	Male participants	Cigarettes/week
F1	0	M1	0
F2	0	M2	0
F3	0	M3	0
F4	0	M4	0
F5	0	M5	0
F6	0	M6	0
F7	0	M7	0
F8	0	M8	0
F9	0	M9	0
F10	0	M10	0
F11	0	M11	0
F12	42	M12	0
F13	42	M13	40
F14	50	M14	40
F15	50	M15	50
F16	50	M16	55
F17	56	M17	60
F18	85	M18	65
F19	100	M19	75
F20	100	M20	85
F21	100	M21	100
F22	140	M22	100
F23	140	M23	105

*Note*. Those who smoked zero cigarettes per week were included in the nonsmoker group.

## Data Availability

The data relative to the study could be obtained by sending an e-mail to fabio.babiloni@uniroma1.it. Prof. Babiloni will return directly the excel file related to the data gathered by the study.
